# Modeling COVID-19 spread in small colleges

**DOI:** 10.1371/journal.pone.0255654

**Published:** 2021-08-18

**Authors:** Riti Bahl, Nicole Eikmeier, Alexandra Fraser, Matthew Junge, Felicia Keesing, Kukai Nakahata, Lily Reeves

**Affiliations:** 1 Mathematics, Bard College, Annandale-on-Hudson, NY, United States of America; 2 Computer Science, Grinnell College, Grinnell, IA, United States of America; 3 Bard College, Annandale-on-Hudson, NY, United States of America; 4 Mathematics, Baruch College, New York, NY, United States of America; 5 Biology, Bard College, Annandale-on-Hudson, NY, United States of America; 6 Applied Mathematics, Cornell University, Ithaca, NY, United States of America; Rutgers The State University of New Jersey, UNITED STATES

## Abstract

We develop an agent-based model on a network meant to capture features unique to COVID-19 spread through a small residential college. We find that a safe reopening requires strong policy from administrators combined with cautious behavior from students. Strong policy includes weekly screening tests with quick turnaround and halving the campus population. Cautious behavior from students means wearing facemasks, socializing less, and showing up for COVID-19 testing. We also find that comprehensive testing and facemasks are the most effective single interventions, building closures can lead to infection spikes in other areas depending on student behavior, and faster return of test results significantly reduces total infections.

## 1 Introduction

Amid Fall 2020 of the COVID-19 pandemic, universities rolled out a variety of interventions in hopes of safely offering in-person instruction [1]. Wrighton and Lawrence argued that “best practices” should be followed, which include: testing, quarantine, contact tracing, facemask usage, and dedensification [2]. While colleges in some parts of the world successfully opened [3], the interventions utilized in the United States were largely untested. A prominent example was the pivot by the University of North Carolina at Chapel Hill to remote instruction after an “untenable” COVID-19 outbreak occurred during the first week of instruction [4]. Other major universities subsequently followed suit in response to similar infection spikes upon reopening [5, 6]. In light of this uncertainty, simulation evidence may help inform policy and guide student behavior.

Some models addressed COVID-19 spread on college campuses [7–11]. We discuss these in more detail in Section 1.4, but note that their primary focus was medium-sized colleges. Given that there are more than 500 colleges in the United States with a student body of 4,000 or less that, in aggregate, serve over a million students, it seems important to specifically address this setting. We develop an agent-based model on a network to simulate COVID-19 spread through a small residential college. The smaller population and campus allow us to make a relatively detailed model. Beyond colleges, we believe that adaptations of our approach could be useful for modeling the effectiveness of interventions in other small, closed-community residential settings such as military bases, single-industry towns, and retirement communities [12, 13].

### 1.1 Base Assumption

Our model contains 2,000 students and 380 faculty. To standardize results, we start each trial with 10 students initially exposed to COVID-19. These agents progress to either the asymptomatic or symptomatic state during which they possibly infect others. The main statistic is the total number of resulting infections after 100 days. Since we seek to compare the effectiveness of different interventions, we require a base model to compare against. There is no data for what would happen during a full semester of regular instruction with unmitigated COVID-19 spread. Our analysis starts with the following assumption.

#### Base Assumption

*Over 80% of the population of a small, residential college would become infected with COVID-19 during a semester with no intervention*.

We stress that our Base Assumption is in the hypothetical situation that no policy and behavior changes occur in response to rising infection counts. Even when a large portion of the population is infected, symptomatic and asymptomatic individuals continue their typical routines: attending class, socializing, and using common spaces on campus as usual. Facemasks are never worn. The administration enacts no mitigating strategies such as: class cancellations, building closures, infection testing and quarantine, contact tracing, and social distancing measures. Complete details about the base model are in Section 2.

We believe that 80% total infections after a semester is conservative given that the population lacks innate antibodies against COVID-19 [14] and the average reproduction number *R*_0_ with no intervention is quite high [15–17] in some settings. Additionally college settings are believed to be worse for COVID-19 spread [18] than in larger communities with less overlap between residents such as cities. Note that a related study [9] predicted that 100% of the campus population would become infected about halfway into a semester with no intervention. More discussion of sensitivity to this choice and difficulties concerning *R*_0_ is in Section 4.

### 1.2 Findings

We use the scenario in the Base Assumption as a control against which we measure the effectiveness of various interventions. Our main findings are given below and discussed further in Section 3.

#### Result 1

*Comprehensive testing and facemask compliance are the most effective single interventions*.

**Fig 1:**
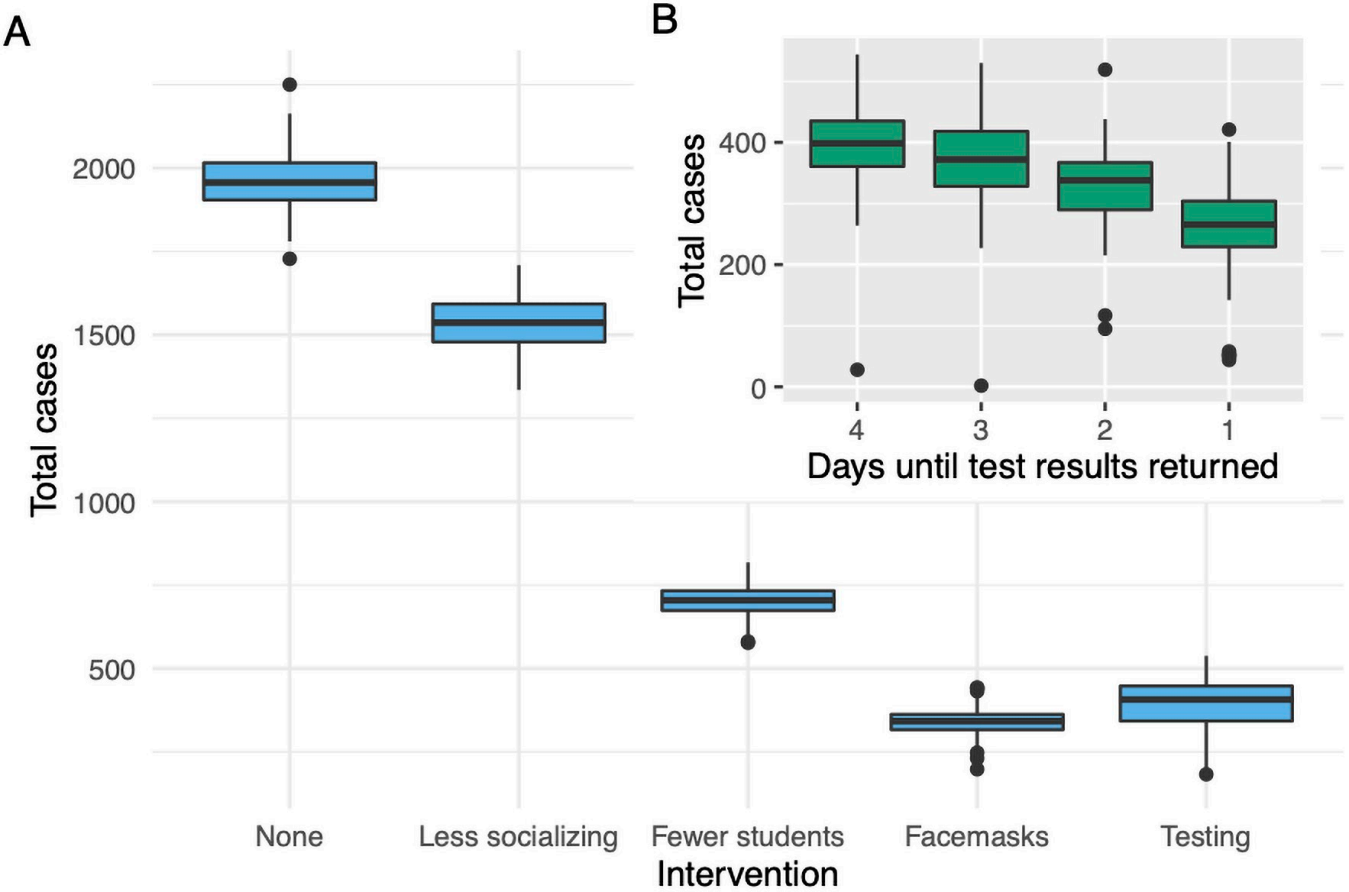
(A) The base model with single interventions applied. Note that the reduction in infections from “fewer students” is smaller than it appears since there are 50% fewer people on campus in that intervention. (B) The impact of testing latency on a campus with 25% fewer students and testing and quarantine in effect.

Weekly COVID-19 screening of 100% of students with a two-day wait for test results brings total infections from around 1,900 to 400 (see Fig 1A). Alternatively, perfect facemask usage in public and social settings drops total infections below 300.

#### Result 2

*Building closures may increase total infections*.

**Fig 2:**
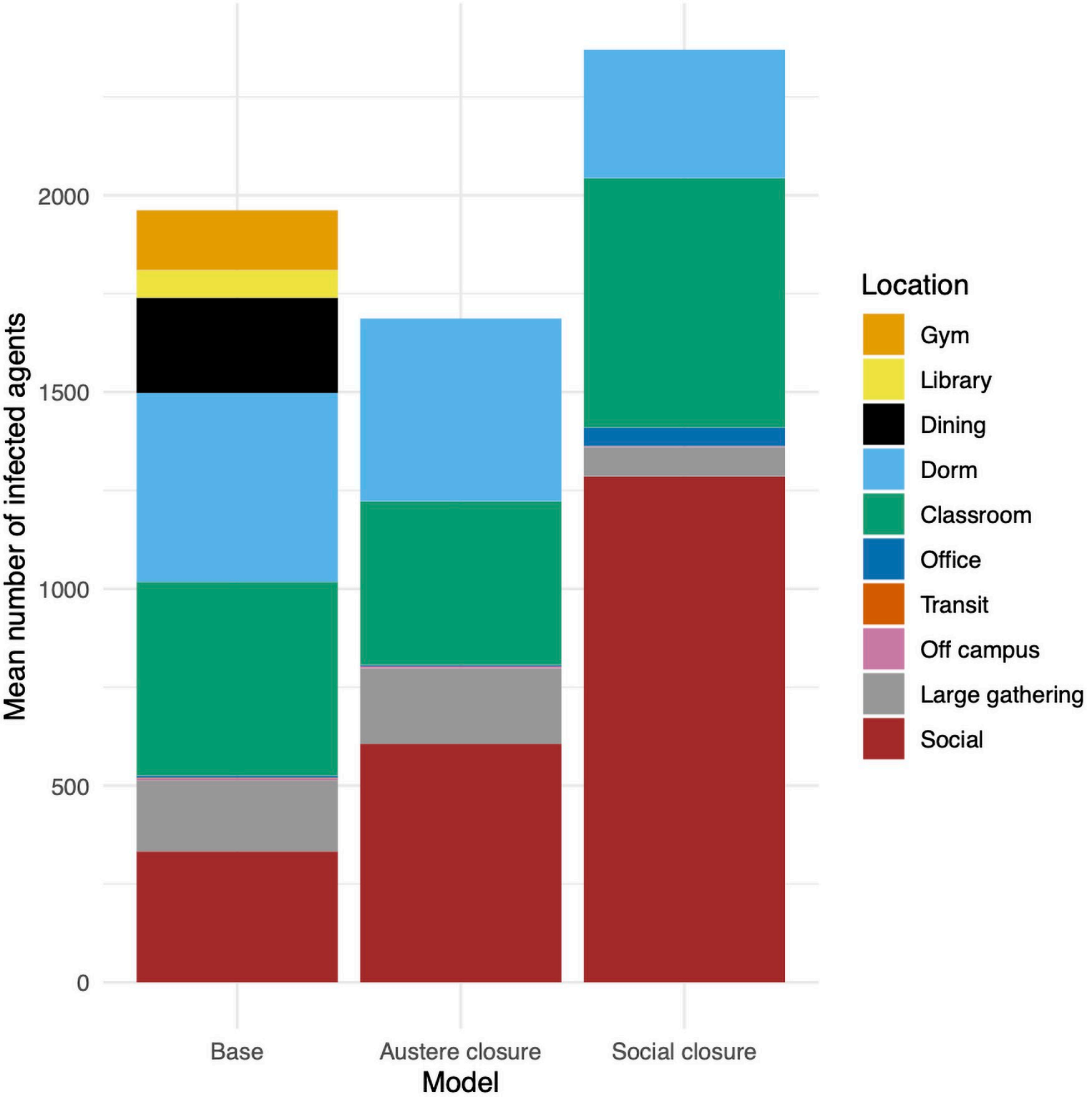
Total infections by room type in the base model and with the gym, library, and dining hall closed. In an “austere closure”, students spend any extra free time alone. In a “social closure”, students spend half of their free time socializing.

Closing the gym, library, and dining hall gives extra unstructured time to students. We find that if students are strict about passing that extra time alone, total infections decrease. However, if students spend half of that time socializing, we see a dramatic spike; nearly every agent in our model becomes infected (see Fig 2).

#### Result 3

*Shortening time to receive test results reduces total infections*.

We consider a campus at 75% density with 50% of students screened weekly for COVID-19 in addition to walk-in testing. No other interventions occur. We then vary the latency period to receive test results from four days down to one. Our model with a four-day latency period results in on average 394 total infections, compared to 259 with a one-day period (see Fig 1B).

#### Result 4

*Strong, unified administrative policy and student adherence result in the best outcomes*.

**Fig 3:**
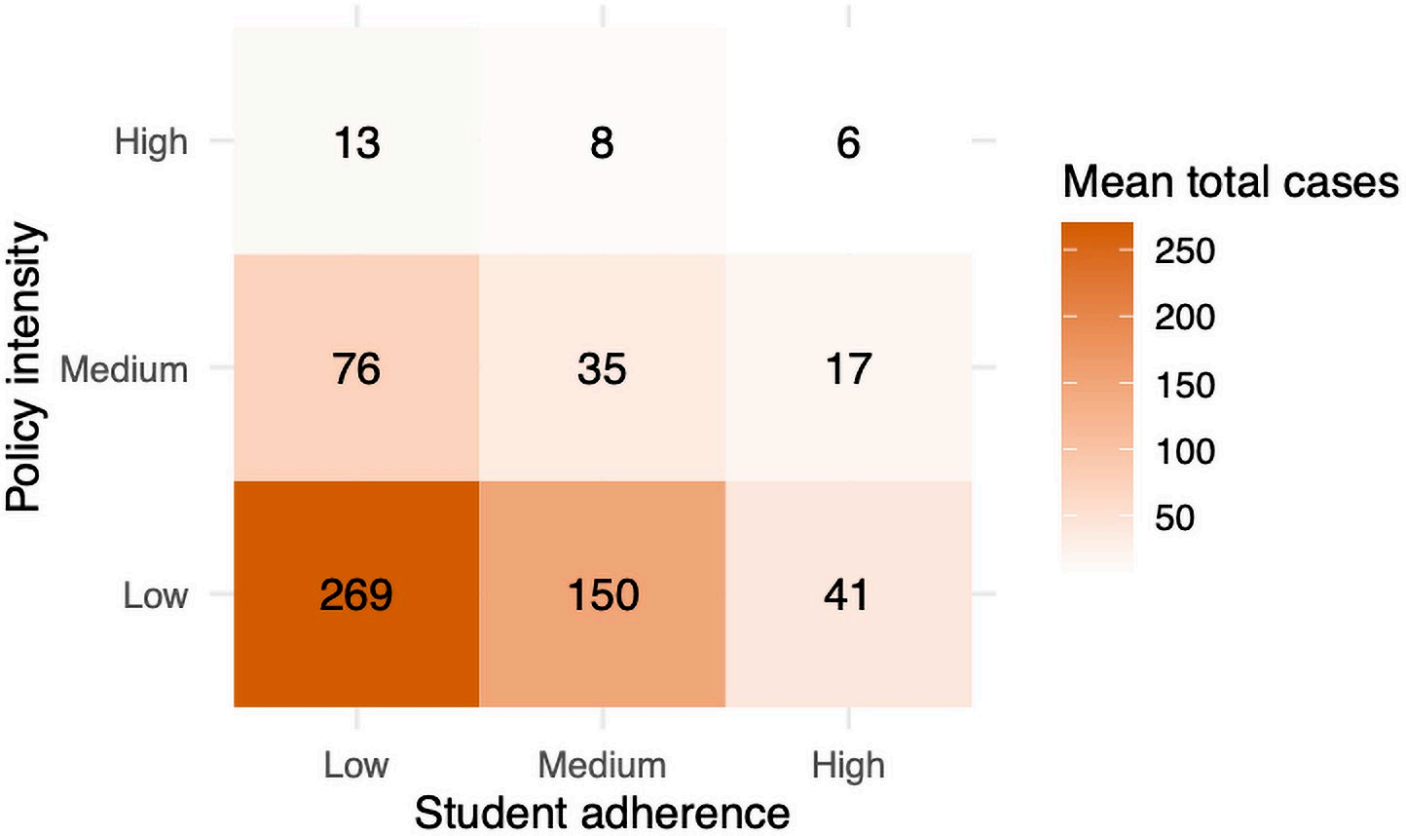
The total infection counts colored by size for different policy and adherence intensities.

A novel part of our intervention design is that we separate student behavior from administrative policy. Specifically, students control facemask usage in social settings, compliance with screening tests, and time spent socializing. Administrators control the number of screening tests, testing latency, building closures, and the number of students allowed back to campus. We consider student adherence and administrative policy at low, medium, and high intensities. A high-intensity administrative policy by itself keeps total infections below 10 with medium levels of student adherence. However, with less intense policy, we find that student adherence plays a crucial role. For example, total infections drop from 269 to 41 as student adherence increases with the low-intensity policy in effect. It is also worth noting that, under a high-intensity administrative policy, there is less variability as a result of student behavior. See Fig 3.

### 1.3 Key takeaways

We outline some possible takeaways for administrators and students.

#### Administrators

Our results suggest that strong administrative policy is needed, particularly regarding testing. Concerned administrators (and students) should check Table 5 to see which intensity their reopening plan most aligns with. We emphasize that the low-intensity policy in our model tests 25% of the student body weekly (Result 4). Without testing at or above this level, our results suggest that it will be hard to control COVID-19 spread. Test latency appears to make a difference as well; we advise that lowering the time to return results be a priority (Result 3). Lastly, we demonstrate that building closures do not necessarily reduce total infections (Result 2). Since social distancing can be more easily controlled in campus buildings, administrators may consider keeping buildings open. At the very least, students displaced by building closures should be encouraged to spend more time in isolation.

#### Students

A serious and disciplined approach is needed from students (and administrators) to keep infections down (Result 4). We recommend that students wear facemasks in private settings, such as socializing, large gatherings, and common space in dorms (Result 1). In light of the increased unstructured time resulting from building closures, it is especially important to spend more time alone rather than socializing (Result 2). Given the impact of testing, students should cooperate fully with any required screening testing (Result 1).

### 1.4 Related work

We know of five projects that specifically addressed COVID-19 spread on a college campus. Gressman and Peck [9] used the University of Pennsylvania as a template to simulate different intervention strategies in an urban university with 22,500 students. This complemented recent work of Weeden and Cornwell [8] that studied how the degree of separation between students at Cornell University changes when some courses are switched to a remote or hybrid format. Around the same time [9] was released, Frazier et al. posted a preprint and, later, an addendum [7] that modeled how testing and quarantine could mitigate the spread of COVID-19 through Cornell’s campus. Recently, Paltiel, Zheng, and Walensky studied the effectiveness of testing in a college with 5,000 students [10]. Durrett et. al developed a mathematical model that rigorously demonstrated the benefits of limiting double occupancy dorms and of capping course enrollments [11].

To briefly summarize [8], showed that a typical student directly interacts with about 4% of the 22, 000 other students from common courses. However, the reach of a student jumps to 87% when considering two degrees of separation, and to 98% with three degrees. The authors further observed that removing large classes with an enrollment over 100 fails to disconnect the network and such interventions only increase the average graph distance between students by about 0.50. For this reason, Weeden and Cornwell recommended taking further action than simply eliminating large courses. The authors also considered liberal arts colleges by restricting to the 4, 500 or so students in Cornell’s College of Arts and Sciences. They observed that students in a liberal arts college are connected via short path lengths, but also through multiple paths. They inferred that this makes ripe social conditions for disease spread.

Frazier et al. also studied the Cornell student body, but rather than considering the network structure, they assumed a perfectly mixed population. They performed an SEIR model primarily taking into account the age of those infected, severity of symptoms, and amount of intervention through testing, quarantine, and contact tracing. They found that such interventions can suppress, but not completely contain the spread of COVID-19 during a semester. Despite fairly heavy intervention, asymptomatic spread results in 1,250 infections in their model. A surprising conclusion drawn from the project was that reopening in the Fall may be *safer* than not reopening. The reason being that many students have commitments and social ties, and would likely return to live in Ithaca during the Fall semester. No campus engagement would increase the amount of unregulated off-campus socializing and ultimately lead to more total cases than in reopening scenarios. theorem 2 demonstrates a similar phenomenon. We further remark that one shortcoming of the approach from Frazier et al. is that the perfect mixing assumption smooths over much of the structure inherent to a campus.

Paltiel, Zheng, and Walensky examined the epidemic outcomes and costs with varying test attributes and epidemic scenarios. They concluded that screening every two days with rapid, inexpensive tests results in a controlled number of infections with relatively low total cost. The authors acknowledged the logistical and financial challenges for university administrators even in the proposed testing scenario. The study did not consider other administrative strategies in combination with testing to restrict the spread of infection.

Gressman and Peck built an agent-based model that incorporated more features of college life. Roughly speaking, on a given day in the model, an agent has approximately 20 contacts selected at random from different groups. These groups included residential, close academic, classroom contact, broad social, etc., and contact came with varying likelihoods of passing an infection. Their results suggested that large scale testing, contact tracing, and moving large classes online were the most impactful interventions. They further found that testing specificity is crucial for managing the number of people in quarantine. The authors observed that their model has limited applicability to small colleges [9, p. 16]. The important difference, in their view, is that students in a small college have fewer, but closer contacts compared to those at a large university. However, they pointed out that, without additional data, the different likelihood of infection may be a “difficult feature to reasonably quantify or calibrate.”

One way we specifically account for social interactions is the introduction of “social spaces” into the network. Each student frequents two social spaces at which they contact a subset of roughly 20 other students. This generates two internally correlated, but externally independent friend groups. More broadly, we draw inspiration from larger agent-based models in which agents diffuse through a to-scale environment according to simple routines [19, 20]. We set the physical network and agent schedules as realistically as possible, then let the academic, residential, and social interactions tune to these choices. This philosophy distinguishes our approach from the models for COVID-19 spread in colleges mentioned above.

## 2 Methods

In this section, we describe the network, agent behavior, and infection dynamics in our base model for a campus with no interventions in place. We conclude by describing different interventions.

Buildings are star graphs whose cores represent shared spaces and leaves represent rooms or sections of the building. Each agent is assigned a fixed schedule that determines their motion through the network which updates hourly (see Table 1). Infection dynamics follow an SEIR model (see (1)) where agents transition from the susceptible to the exposed state with probability proportional to the number of nearby infected agents scaled by the riskiness and size of the space (see (2)). We set the parameters (see Tables 2–4) to reflect the unique features of a small college campus—small classes; tightly knit, but diverse social groups; a primary dining hall, gym, and library—as well as our present understanding of the biology of COVID-19. We then overlay various interventions on the base model and measure their effectiveness.

**Table 1:** Sample schedules for an on-campus student, an off-campus student, and a faculty member. Each row is the time of day.

	**On-Campus**			**Off-Campus**		**Faculty**	
	*A*	*B*	*W*	*A*	*B*	*A*	*B*
8	D	D	D				
9	DH	D	DH	OC	OC	OC	OC
10	*C*_1_	DH	D	*C*_1_	L	O	O
11	*C*_1_	S	L	*C*_1_	S	O	O
12	DH	*C*_4_	S	DH	*C*_4_	DH	O
13	S	*C*_4_	DH	L	*C*_4_	O	DH
14	*C*_2_	DH	S	*C*_2_	DH	*C*_1_	*C*_2_
15	*C*_2_	G	G	*C*_2_	G	*C*_1_	*C*_2_
16	*C*_3_	D	L	*C*_3_	L	O	O
17	*C*_3_	S	L	*C*_3_	S	O	O
18	DH	D	D	OC	OC	OC	OC
19	L	DH	DH				
20	S	D	S				
21	D	D	S				
22	D	D	D				

**Key**	
D	Dorm
DH	Dining Hall
*C*_*i*_	*i*th class
S	Social Space
L	Library
G	Gym
OC	Off Campus
O	Office

**Table 2:** At the top, counts for the number of single and double dorm rooms, the number of seats in classrooms. In the middle, the number of classrooms in each type of building. On the bottom, the number of each type of building.

	Single	Double	Smls	Mds	Lrgs	Seats	Capacity
Small Dorm	5	5					15
Medium Dorm	15	15					45
Large Dorm	25	25					75
Small Clsrm						10	15
Medium Clsrm						15	20
Large Clsrm						20	30
Small Acad			3	0	0	30	45
Medium Acad			2	3	0	65	90
Large Acad			5	3	3	155	225
Dorm Bldgs			25	10	10		1575
STEM Bldgs			2	2	3	655	945
Humanities Bldgs			1	2	1	315	450
Arts Bldgs			2	1	1	280	405

**Table 3:** The core and leaf capacity and risk multiplier for different buildings. The quantity *x* is the number of people assigned to that space.

Space	Core		Leaf	
	*C*_*v*_	*r*_*v*_	*C*_*v*_	*r*_*v*_
Transit Space	100*n*	1		
Dining Hall	650	1	100	2
Faculty Dining Leaf			20	2
Library	10 ⋅ 300	1	50	2
Gym	10 ⋅ 60	3	10	3
STEM Office	10 ⋅ 6 ⋅ 50	1	50	2
Hum/Art Office	10 ⋅ 6 ⋅ 25	1	20	2
Social Space			10	3
Large Gatherings	40⌈*x*/40⌉	3		
Small Acad	10 ⋅ 45	1		
Medium Acad	10 ⋅ 90	1		
Large Acad	10 ⋅ 225	1		
Small Clsrm			15	2
Medium Clsrm			20	2
Large Clsrm			30	2
Single Dorm			1	3
Double Dorm			2	3
Small Dorm	10 ⋅ 15	2	*x*	3
Medium Dorm	10 ⋅ 45	2	*x*	3
Large Dorm	10 ⋅ 75	2	*x*	3

**Table 4:** Parameters.

Parameter	Value	Description	Ref
**Base Model**			
$\left(n_{c};n_{c}^{1},n_{c}^{2},n_{c}^{3}\right)$	(1500; 750, 375, 375)	on-campus student counts by division	[27]
$\left(n_{o};n_{o}^{1},n_{o}^{2},n_{o}^{3}\right)$	(500; 250, 125, 125)	off-campus student counts by division	[27]
$\left(n_{f};n_{f}^{1},n_{f}^{2},n_{f}^{3}\right)$	(380; 190, 95, 95)	faculty counts by division	[27, 28]
(*g*, *s*, *ℓ*)	(0.15, 0.15, 0.15)	gym, social, and library probabilities	[29, 30]
*o*	0.125/(*n*_*o*_ + *n*_*f*_)	off-campus infection probability	
*T*_*E*_	2	days in the exposed state	[31]
*a*	0.15	probability of remaining asymptomatic	[32]
*e*	0.50	probability of *I*^*a*^ → *I*^*e*^	[33]
$T_{I^{a}}$	10	days in *I*^*a*^ if asymptomatic	
$T_{I^{a}}^{*}$	2	days in *I*^*a*^ if symptomatic	[34]
$T_{I^{e}}$	10	days in *I*^*e*^ if never bid-ridden	[15]
$T_{I^{e}}^{*}$	5	days in *I*^*e*^ if bed-ridden	[15]
$T_{I^{m}}$	10	days in *I*^*m*^	[35]
*p*	1.25	tuning parameter	
*FP*	0.001	false positive rate	[9]
*FN*	0.03	false negative rate	[9]
**Interventions**			
f	0, 0.50, 1	facemask compliance	
m	0.50	facemask reduced infectiousness	[21–26]
$m^{\prime }$	0.75	facemask protection from infection	[21–26]
P	0.20, 0.50, 1	weekly percentage of students screened	
L	1, 2, 3, 4	latency period to receive results	
c	0.80, 0.90, 1	asymptomatic screening compliance	
*q*_*e*_	0.95	probability of symptomatic walk-in test	
*q*_*m*_	0.70	probability of mild walk-in test	
*FP*	0.001	false positive rate	[9]
*FN*	0.03	false negative rate	[9]
B	*L*, *G*, *DH*, *O*, *LG*	building closures	[36]
h	0.50, 0.75, 1	prob. of dorm/off-campus from bldg. closure	
D	0, 650, 1300	dedensification amount	[37]
s	0, 0.25, 0.75	reduction in socializing	
i	5, 7, 10	initial infected cases with dedensification	

### 2.1 Space

**Fig 4:**
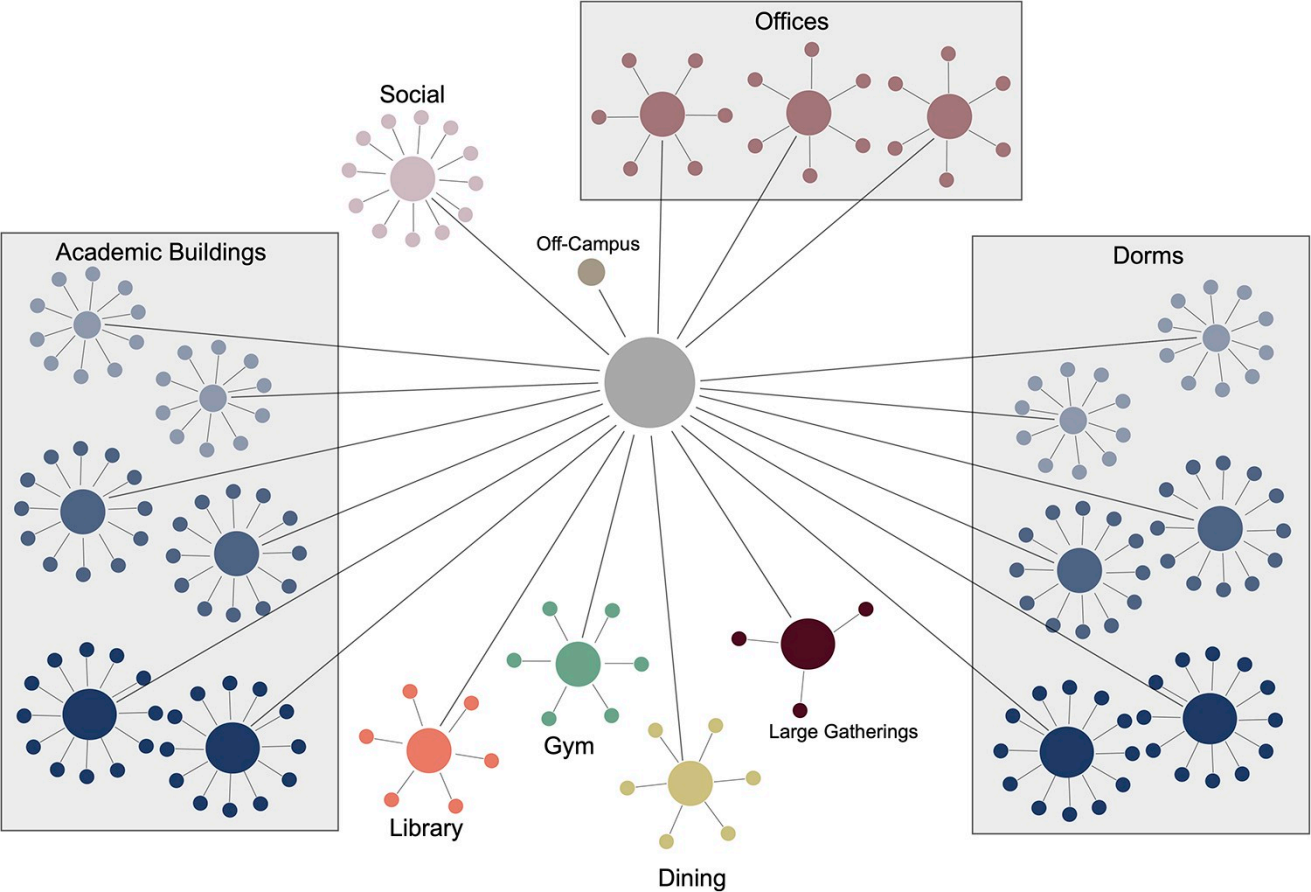
Schematic of the network.

Many of our decisions regarding our network draw inspiration from the campuses of Bard College and Grinnell College which exemplify small, relatively isolated, residential colleges. The basic building blocks are star graphs representing dorms, academic buildings, dining halls, gyms, social spaces, offices, and off-campus. The core of each star represents shared space in the building such as hallways, bathrooms, lobbies, etc. The leaves represent either specific rooms or sections of the building. See Table 2 for specifics. The core of each star connects to the transit vertex which represents the connective space between buildings. Note that the graph diameter is 4. See Fig 4 for a schematic.

#### Dorms, classrooms, academic buildings

Are either small, medium, or large depending on the number of single and double rooms (Dorms), the number of seats (Classrooms), or the number of classroom sizes (Academic Buildings).

#### Dining hall, gym, library, faculty offices

Are modeled by star graphs with six leaves. The leaves represent sections of the buildings. Our network has one gym, one library, one dining hall, and three faculty offices.

#### Social spaces

Are leaves of a star graph. The spaces represent social gatherings (study sessions, work groups, parties, casual social groups) that occur at various locations on campus. There are 100 such leaves. The core has no meaning, but is included for the sake of consistency in the underlying network.

#### Transit space

Is a single vertex that represents the paths, halls, and rooms that connect the other spaces.

#### Off campus

Is a single vertex that represents all space off campus.

### 2.2 Agent behavior

In this section, we describe the types of agents, the way they are assigned schedules, and how they move through the network.

#### Agent types

There are *n* = 2, 380 total agents in the model; with *n*_*c*_ = 1, 500 on-campus students, *n*_*o*_ = 500 off-campus students, and *n*_*f*_ = 380 faculties. Agents are assigned a subtype that designates their division among STEM, Humanities, and Arts. We write $n_{*}^{i}$ with *i* = 1, 2, 3 and * ∈ {*c*, *o*, *f*} to denote the counts of STEM (*i* = 1), Humanities (*i* = 2), and Arts (*i* = 3) agents. We assume that STEM students are 50% of the student body, Humanities students are 25%, and Art students are 25%. Note that the division designations are interchangeable so these proportions represent whatever specialty a small college may have.

#### Agent schedules

Days are classified as either *A*, *B*, *W*, or *S*. *A* and *B* days are distinguished by alternating class schedules. *W* days represent weekends (Friday and Saturday) on which no instruction occurs and students socialize. To introduce some space into schedules, we include Sundays (*S*) on which students either stay in their dorms or off-campus all day. A day is divided into 14 one-hour increments spanning from 8:00—22:00 (the time *N*:00 will be abbreviated by *N*). Classes take place in two-hour increments starting at 10, 12, 14, and 16.

We write each seat in a class on a given day and time as a 4-tuple (*d*, *t*, *r*, *c*) where *d* ∈ {*A*, *B*}, *t* ∈ {10, 12, 14, 16}, *r* is a classroom, and *c* is a chair in *r* (so 1 ≤ *c* ≤ the enrollment capacity of room *r*). Let C be the set of all distinct seats (*d*, *t*, *r*, *c*). Let $C_{1}$ denote the set of all tuples whose building is designated a STEM building, and similarly for $C_{2}$ and $C_{3}$ for Humanities and Arts, respectively. Let $C=C_{1}\cup C_{2}\cup C_{3}$. To randomly assign classes, students with subtype *i*, one after the other, sample two elements uniformly at random from $C_{i}$ and then two elements uniformly at random from C without replacement. If two selections conflict in time, classrooms are resampled until there are no conflicts.

Once an agent obtains a class schedule, the remaining time slots are filled in according to the following rules. For each building in the schedule that is not a dorm or academic building, the agent is assigned to a uniformly sampled leaf, which they exclusively visit. The one exception concerns social spaces. For these, students are assigned a leaf for class days, and a leaf for the weekend. Since there are 100 social space leaves, on average 20 students are assigned to each leaf. Being assigned to two leaves makes it so agents interact with two social groups that are correlated within, but uncorrelated to other groups.

For *on-campus students*, each day begins and ends in their assigned dorm room at 8 and 22. Up to two students may be assigned to a given dorm room, which corresponds to having a roommate. Each day type has one visit to the dining hall in the time slots 8–11, 12–15, 17–20. The afternoon slot 12–15 is skipped if the student has classes during that time. Lastly, each day type has a gym visit with probability *g*. The remaining slots are assigned to uniformly sampled social spaces with probability *s*, a library leaf with probability *ℓ*, or the agent’s assigned dorm room with probability 1 − *s* − *ℓ*.

For *off-campus students*, *A* and *B* days begin and end at the Off Campus vertex at times 8, 9 and 18–22. On *W* and *S* days the student remains at the Off Campus vertex all day. On *A* and *B* days, an off-campus student has one visit to the dining hall in the time slots 12–15, if the class schedule allows it. Each day type contains a gym visit with probability *g* at a randomly chosen available time slot. The remaining slots are spent in a social space with probability *s*, at the library with probability *ℓ*, and otherwise off-campus.

For *faculty*, *A* and *B* days begin and end with the agent at the Off Campus vertex at times 8, 9 and 18–22. On *W* and *S* days the faculty remains at the Off Campus vertex all day. If possible, the agent goes to the faculty leaf of the dining hall at a uniformly chosen time from 11–13. The remaining slots are spent in the appropriate Division Office vertex.

#### Agent paths

Once an agent is assigned a schedule it remains to define the path the agent follows to move between each location. Suppose an agent is moving from a leaf of the core vertex *v* to a leaf of the core vertex *u*. They do so by moving to *v*, to the transit vertex, to *u*, and then to the target leaf of *u*. We assume that transit occurs at the end of the hour and interacts with any other agents that move through the spaces *u*, the transit vertex, and *v* at the end of the same hour.

### 2.3 Infection spread

#### Agent states

Agents are in states *S*, *E*, *I*^*a*^, *I*^*m*^, *I*^*e*^ and *R* corresponding to Susceptible, Exposed, Infected Asymptomatic, Infected Mildly Symptomatic, Infected Extremely Symptomatic, and Recovered. Agents transition through the states in the following manner:

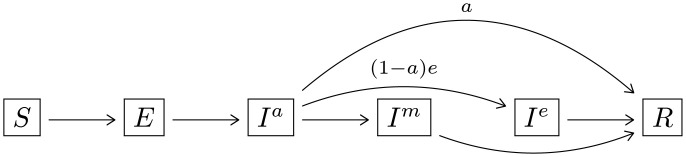
(1)
We let $I_{v}^{a}\left(d,t\right)$ denote the number of agents in state *I*^*a*^ at site *v* at time (*d*, *t*) and similarly for the other states. Describing how and when agents transition from state *S* to state *E* is the subject of the next section. The other transitions are simple to describe:

- Agents stay in state *E* for *T*_*E*_ = 2 days. After which, they transition to state *I*^*a*^.
- Each agent in state *I*^*a*^ transitions to state *R* after $T_{I^{a}}=10$ days (from the day of infection) with probability *a*. Otherwise, after $T_{I^{a}}^{*}=2$ days the agent transitions to state *I*^*e*^ with probability *e* and to state *I*^*m*^ with probability 1 − (*a* + *e*).
- Each agent in state *I*^*e*^ transitions to state *R* after $T_{I^{e}}=10$ days. However, after $T_{I^{e}}^{*}=5$ days the agent spends the subsequent time in their dorm room. This represents a student becoming “bed-ridden,” i.e., too sick to leave their room.
- Agents in state *I*^*m*^ transition to state *R* after $T_{I^{m}}=10$ days.

#### The base probability of infection

The vertex *v* at time (*d*, *t*) has *infection probability*

$$\begin{array}{c} p_{v}\left(d,t\right)=r_{v}\frac{I_{v}^{e}\left(d,t\right)+I_{v}^{m}\left(d,t\right)+0.5I_{v}^{a}\left(d,t\right)}{C_{v}}p. \end{array}$$ (2)

The parameter *C*_*v*_ is the *capacity* of *v* and *r*_*v*_ ∈ {0, 1, 2, 3} is the *risk multiplier* for infection spread in that space. Each of the *S*_*t*_(*v*) susceptible agents at *v* at time *t* independently enters state *E* with the probability at (2). Note that we set the infectiousness of an agent in state *I*^*a*^ to half that of an agent in the other infected states [21]. The constant *p* is the *tuning parameter* that allows us to control global infectiousness.

#### The risk and capacity parameters

The parameter *r*_*v*_ is chosen based on time spent, the proximity of agents in the space, and the typical amount of respiration—i.e. time spent talking aloud or exercising—in a given space. For example, *r*_*v*_ is higher in the gym compared to the library. We set *C*_*v*_ equal to ten times the core capacity for buildings with known capacities in advance (dorms and instructional buildings). The factor of ten is to dilute the number of people in the core at a given time (otherwise all of the agents would simultaneously be in that location). Ten is chosen since a passing time between classes is about that duration in minutes. The capacities for the dining hall, library, gym, and social spaces are set empirically to match the typical occupancy of the building. See Table 3 for all of the *C*_*v*_ and *r*_*v*_ values.

#### Exceptions

Two exceptional spaces, where the infection dynamics are not exclusively governed by (2), are off-campus and large gatherings. Upon leaving the off-campus vertex at *t* = 8, each agent in state *S* transitions to state *E* with probability *o*. For agents *returning from off-campus*, we choose *o* = .125/(*n*_*o*_ + *n*_*f*_) so that, on average, one off campus agent becomes infected every 8 class days (two weeks). For *large gatherings*, half of the student agents (both on- and off-campus) are denoted as “social.” We simulate large informal gatherings (e.g., parties or organized social events) by drawing three random subsets *G*_1_, *G*_2_, *G*_3_ of agents designated as social at the end of each week. Each *G*_*i*_ has size uniformly and independently sampled from [20, 60]. The *G*_*i*_ are sampled independently and are not necessarily disjoint. Each susceptible agent at a large gathering becomes infected according to (2) with *r*_*v*_ = 3 and *C*_*v*_ = 40⌈|*G*_*i*_|/40⌉, i.e., *C*_*v*_ = 40 if |*G*_*i*_| ≤ 40, and *C*_*v*_ = 80 if |*G*_*i*_| > 40.

### 2.4 Contact structure

Section 2.1 describes the campus network. Agents move through this network by following hourly schedules generated according to the specification in Section 2.2. We then overlay COVID-19 spread according to the rules in Section 2.3. The likelihood of infection spread is given at (2), and ultimately governed by the risk factor and capacity of each site in the network. We measure the aggregate exposure between agents by summing the risk scaled by the capacity over all of an agents interactions during a simulated week in the model.

More precisely, given an agent *i*, we generate a vector ${\vec{e}}_{i}=\left(e_{i,1},\ldots ,e_{i,N}\right)$ where

$$\begin{array}{c} e_{i,j}=\sum_{\left(d,t,v\right)\in S_{i}}1\left\{\text{agent}\;j\;\text{also}\;\text{at}\;v\;\text{on}\;\text{day}\;d\;\text{at}\;\text{time}\;t\right\}\frac{r_{v}}{C_{v}} \end{array}$$

with *S*_*i*_ the set of vertices that *i* visits over the course of one week. So we sum the risk factor scaled by the capacity of all of the vertices that *i* interacts with *j* at. We call the vector ${\vec{e}}_{i}$ the *exposure profile* of agent *i* with the individual entries *e*_*i*,*j*_ the *exposure level* of agent *i* to agent *j*. Note that *e*_*i*,*j*_ = *e*_*j*,*i*_ be symmetry of the model.

**Fig 5:**
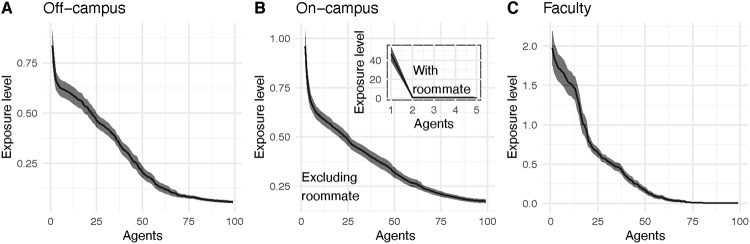
Exposure profiles for 100 agents are arranged in decreasing order then averaged. A 95% confidence interval is included around the curve. Panel A shows the exposure profile for off-campus students. The larger panel of Panel B shows the exposure profile for on-campus students with the maximum entry (corresponding to a dorm roommate) removed. The smaller subpanel in Panel B shows the exposure profile when the roommate is included. Panel C shows the ordered average exposure profile for 100 faculty.

To generate Fig 5 we sampled the exposure profiles of 100 on-campus, 100 off-campus, and 100 faculty agents. The exposure levels were then arranged in decreasing order. For on-campus students with a roommate we throw out the first entry since it is on a different order than the others. This represents the feature of our model that roommates are most likely to infect one another. We then plotted a curve representing a 95% confidence interval around the mean level of each entry for each agent type. We observe that agents have high exposure levels with ten or so other agents and the exposure level drops roughly linearly until about 50 to 75 agents. Subsequently, the exposure level is low with the remaining 2300 agents.

This data suggests that the contact structure of our network is such that each individual has ten or so close contact with whom they are likely to spread infection. These high exposure levels are coming from socializing and faculty interactions in their departmental buildings. Agents with medium exposure levels (in the interval [25, 75]) come from classroom contact and exposure in dorm common spaces. The rest of the campus population has small exposure levels. This heterogeneity of exposure profiles suggests that our model is more nuanced than commonly used homogeneously mixing SEIR models in which all exposure levels would be equal.

### 2.5 Types of intervention

We consider a variety of interventions that broadly include: facemasks, testing/quarantine, building closures, less socializing, and dedensification, which we describe in more detail below.

#### Facemasks

We assume that agents never wear facemasks at dorm and dining hall leaves. There is partial compliance at dorm cores, social space leaves, and large gatherings. All other vertices have perfect compliance. Let f∈{0.50,1} be the proportion of compliant agents. We implement this intervention by randomly selecting the corresponding percentage of agents who always wear a facemask at partial compliance vertices. We assume that wearing a mask reduces an agent’s infectiviousness by a factor of m=0.5 (which is the conservative estimate from [21] and in line with other estimates from [22–26]). So, an infected agent wearing a mask is a factor of m less infectious, and a susceptible agent wearing a mask is a factor of $m^{\prime }=0.75$ less likely to become infected at each time location. That facemasks protect the wearer (although to a lesser extent than the reduction in infectiousness from an infected agent wearing a mask) from inhaling the virus is supported by evidence from [22, 26]. For example, a susceptible person wearing a mask in room *v* at time (*d*, *t*) will become infected with probability

$$\begin{array}{c} p_{v}^{\prime }\left(d,t\right)=m^{\prime }\frac{mM_{v}\left(d,t\right)+I_{v}\left(t,d\right)}{C_{v}}p \end{array}$$ (3)

rather than (2), where $M_{v}\left(d,t\right)=M_{v}^{e}\left(d,t\right)+M_{v}^{m}\left(d,t\right)+0.50M_{v}^{a}\left(d,t\right)$ are the number of agents in the infected state wearing a mask at *v* at time (*d*, *t*) and $I_{v}\left(d,t\right)=I_{v}^{e}\left(t\right)+I_{v}^{m}\left(d,t\right)+0.50I_{v}^{a}\left(d,t\right)$ are (weighted by infectiousness) number of infected agents in the infected state not wearing a mask at *v* at time (*d*, *t*).

#### Testing and quarantine

In line with [9], we assume a false positive rate of *FP* = 0.001 for agents tested while in the susceptible or exposed state, and a false negative rate of *FN* = 0.03 for agents tested while in an infected state.

*Screening*. We assume that P∈{0.25,0.50,1} of the student body is screened per week. Only students are screened, and the screening is applied throughout the entire student body on a repeating cycle. The *latency period* L∈{1,2,3,4} is the number of days to receive results. After the latency period, the infected agents from the batch who test positive are placed in the quarantine state for 14 days, after which they transition to the recovered or susceptible state depending on whether or not the test was correct. We consider c∈{0.80,0.90,1} the level of compliance for agents in state *I*^*a*^ to get screened. This means that each time an agent in the *S*, *E*, or *I*^*a*^ state is selected for screening, the agent skips taking the test with probability 1-c.

*Walk-ins*. For each day following the first that an agent enters state *I*^*e*^ or *I*^*m*^, that agent opts to be tested with probabilities *q*_*e*_ = 0.95 and *q*_*m*_ = 0.70. After this, the agent enters the quarantine state with probability 1 − *FN* depending on if they are in state *I*^*m*^, *I*^*e*^, or *I*^*a*^. For example, the probability an agent in state *I*^*e*^ enters the quarantined state *k* days after entering state *I*^*e*^ is (1 − *FN*)(1 − *q*_*e*_)^*k*−1^ *q*_*e*_. The probability q* represents an agent ignoring symptoms on a given day and waiting to take the test. We assume that walk-ins immediately begin quarantine, but re-enter the campus if they receive a false negative result.

#### Closures

We assume that buildings in B⊆{L,G,DH,O,LG} are closed. If the library (*L*), gym (*G*), or dining hall (*DH*) are closed, time spent at the space is replaced in a student’s schedule with time in the student’s dorm room or off-campus, depending on the type of student, with probability h∈{0.50,0.75,1}. Otherwise, the agent goes to the social space. When facing a building closure, faculties spend that time in their office instead. When faculty offices (*O*) are closed, no infection occurs there, and we assume faculty only spend time in the classes they teach. When large gatherings (*LG*) are removed, we turn off the large gathering component.

#### Dedensification

For *medium dedensification* we remove D=650 agents: 250 on-campus, and 250 off-campus students, as well as 150 faculty at random. For *high dedensification* we remove 1300 agents: 500 on-campus students, 500 off-campus students, and 300 faculty from the campus. The first students to be removed are those in double rooms.

A few technicalities emerge with dedensification in effect. Courses in either degree of dedensification are assumed to be hybrid. All classes continue to meet, but the removed students attend class remotely. We assume that large gatherings do not occur whenever dedensification is in place. Lastly, a dedensified campus will naturally have fewer initially infected agents. We account for this by starting with i∈{5,7,10} on-campus students infected, with i chosen to be approximately 0.05% of the students and faculty still utilizing the campus. When D=650, we assume that i=7, and when D=1300 we assume that i=5.

#### Less socializing

We replace time in social spaces with time spent at the student’s dorm room or the off-campus vertex depending on the type of student. This replacement is done to each occurrence of social space in an agent’s schedule with probability s∈{0,0.25,0.75}.

At this point we have defined all of the parameters in our model. Table 4 summarizes these choices.

## 3 Results

There are over a hundred thousand distinct combinations of the five single interventions from Section 2.5. Therefore, some care is required to decide what combinations provide useful insights. To this end, we reduce down to 20 strategies and focus on *total infections*. This is the total number of agents ever in the exposed state after running the model for 100 days with i on-campus students initially in the exposed state. The value of i∈{5,7,10} depends on the amount of dedensification and is not counted towards total infections. We perform 40 independent simulation trials for each model (with new schedules in each trial). Each trial takes a little over a minute to simulate on a home computer. It takes about a day on a single machine to run all of the interventions described below.

### Marginals

**Fig 6:**
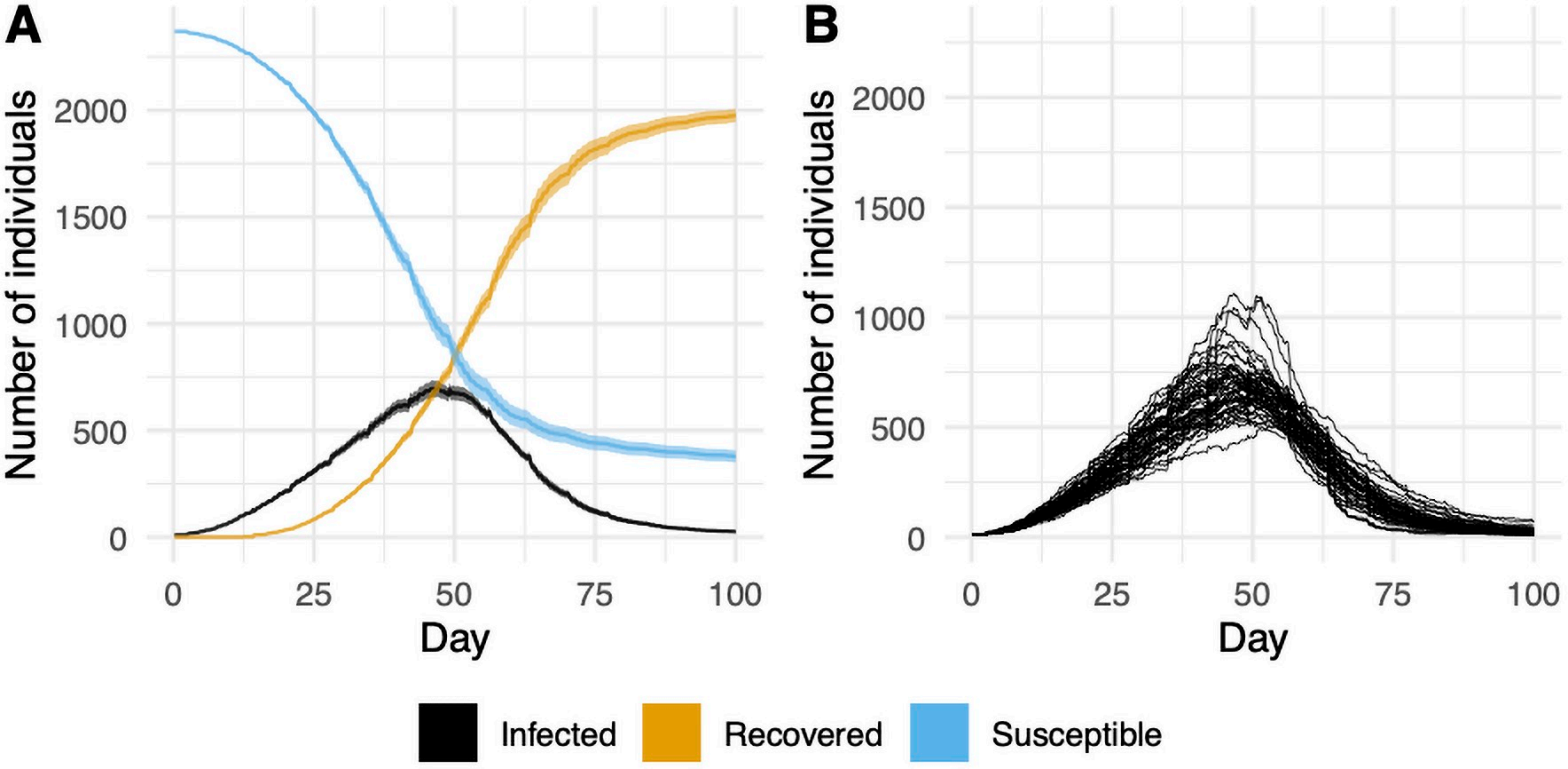
Agent states over 100 days in the base model. Panel A shows a 95% confidence around the mean behavior from 40 trials. Panel B shows the number of active infections over time for each trial.

We apply single interventions at high-intensity to the base model. Specifically, we consider: no intervention, facemasks with f=1, high dedensification with D=1300, less socializing with s=0.75, and testing with P=1. The results are shown in Figs 1, 2 and 6.

### Building closures

We close the gym, libarary, and dining hall with h=0.50 and h=1. No other interventions are applied. See Fig 2.

### Test latency

We fix the base model with medium dedensification (D=650) and testing with P=0.50. This means that there are about 25% fewer students on campus, of whom 50% are screened weekly. We then consider latency L∈{1,2,3,4}. The results are shown in Fig 1.

### Policy and adherence

**Fig 7:**
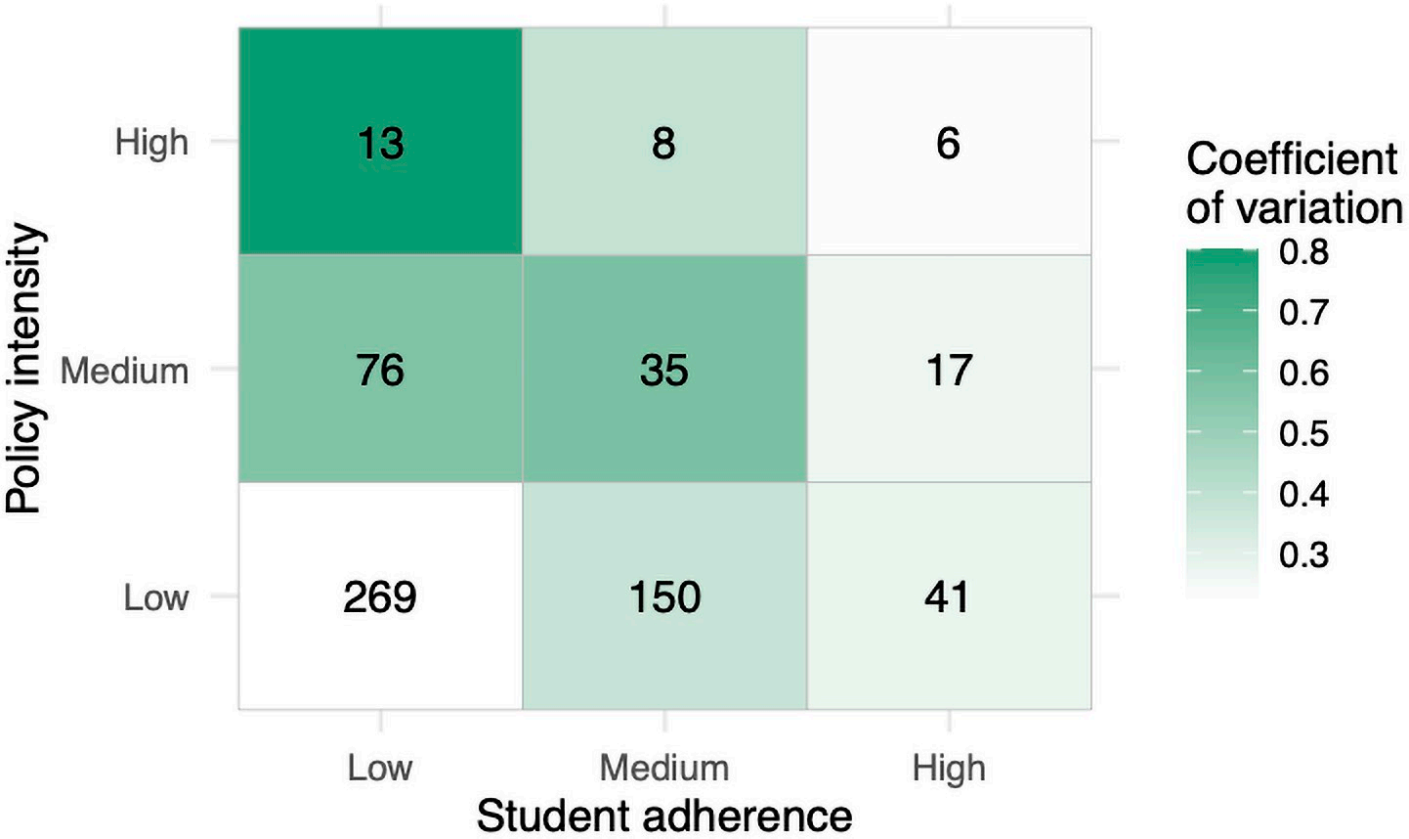
The total number of cases (numeric) and the coefficient of variation (standard deviation/mean; colorbars) for different policy and adherence intensity levels.

To address the problem of choosing which interventions to run among the many we could apply, we classify the single interventions as either an administrative policy, or a student adherence behavior. We group interventions by type and set each to one of three different intensity levels. This gives nine combined strategies, which we hope offer a practical perspective for students and administrators attempting to manage the risk of COVID-19 spread. The specific parameters used for low, medium, and high-intensity policy/adherence are given in Table 5. Administrators control the amount of testing P, test latency L, the amount of dedensification D, and building closures B. Students control facemask adherence f, testing compliance c, how they spend time that would normally be spent in a closed building h, and how much they reduce socializing s. The results are shown in Figs 3 and 7.

**Table 5:** The intervention parameter choices corresponding to different intensities for administrative policy (left) and student adherence (right). We describe in words Medium Policy and Medium Student Adherence as an example. Medium Policy screens P=0.50 of the student population weekly with a 3-day latency L. D=650 students are removed from the population. The gym, library, dining hall, and large gatherings are closed. Medium student adherence has half of students wearing facemasks while socializing f=0.50. A c=0.90 proportion of students comply with screening tests. Students spend free time from building closures in their dorm room with probability h=0.75 for each occurrence in their schedule. Additionally, students socialize less by a factor of s=0.25.

	Policy				Adherence			
	P	L	D	B	f	c	h	s
Low	0.25	4	0	{*G*, *L*}	0	0.80	0.50	0
Medium	0.50	3	650	{*G*, *L*, *DH*, *LG*}	0.50	0.90	0.75	0.25
High	0.75	2	1300	{*G*, *L*, *DH*, *O*, *LG*}	1	1	1	0.75

Recall, that our primary findings are:

1. Comprehensive testing and facemask compliance are the most effective single interventions.
2. Building closures may increase total infections.
3. Shortening time to receive test results reduces total infections.
4. Strong, unified administrative policy and student adherence result in the best outcomes.

We now explain how these experiments support these results.

### Base model

In our base model, we set the tuning parameter *p* = 1.25. This consistently leads to a large infection that reaches on average 1988 agents (see Fig 1). Fig 6 displays the evolution of the infection over time. The peak typically occurs between 40 and 50 days into the semester. Fig 6 A shows two standard deviations of data. The breakdown of infection counts by building type are given in Fig 2. Dorms, classrooms, social spaces, and the dining hall make up the majority of cases. Large gatherings and the gym are next.

### Result 1

Fig 1 shows how weekly testing of 100% of students with latency at L=2, consistently reduces infections below 400. With facemask usage, total infections stay around 300 (Fig 1). Note that Fig 1 is somewhat misleading in its depiction of the effectiveness of high dedensification (the “fewer students” box), because there are only half as many agents present during that intervention.

### Result 2

Fig 2 shows the vertices where infections occur in the base model alongside the effects of closing the gym, library, and dining hall. With closures, we consider the settings with h=1 and h=0.50. We call the case h=1 an “austere closure” since students are electing to pass the time slots they would have been in a closed building at either their dorm room or off-campus. With an austere closure, total infections drop from nearly 2000 to around 1700. The total number of infections in social spaces increases, since these infections would normally occur earlier in a closed building, but instead occur later in a social space. The case h=0.50 is a “social closure” in which students go to social spaces with probability 0.50. The last column of Fig 2 shows a significant increase in infections. A huge increase in social space infections allows the infection to proliferate. We note that the final counts are unrealistic, since it seems unlikely to us that a college would remain open after so many students are infected. Nonetheless, the mixed effect of closing buildings is illustrated by these counts.

### Result 3

As L goes from 4 to 1 total infection counts drop from 394 on average to 259. See Fig 1B. One interesting feature is that the variance increases as L decreases. When L=4, the standard deviation in total infections is 60; but when L=1, the standard deviation is 87. The reason for the greater volatility is that shorter latency sometimes is very effective and completely controls the infection, and sometimes the infection spreads more quickly than testing can control, resulting in many infections (relative to the mean).

### Result 4

Fig 3 shows that the average number of total infections drops from 269 to 6 as policy and adherence are strengthened. The standard deviation drops significantly as well. We see that total infections are reasonably controlled by high-intensity policy (top row of Fig 3). Fig 7 displays the coefficient of variation (standard deviation/mean). The figure illustrates how low-intensity policy coupled with low adherence, even after normalizing for the mean, has the highest variation. Additionally, Fig 7 shows that high-intensity administrative policy can temper variation stemming from different levels of student adherence.

## 4 Discussion

### 4.1 The average reproduction number

The *average reproduction number R*_0_ is the mean number of direct infections originating from a single infected agent in a completely susceptible population. This assumes no preventative measures are being taken. Compare to *R*_*t*_ which measures the mean number of infections at a given point in time as interventions occur and immunity develops in the population. The emerging consensus is that the value of *R*_0_ particular to COVID-19 lies in the interval [2, 3] [17]. However, estimates vary [15, 16], and as put by [38] “estimates of *R*_0_ in one population do not necessarily translate to another.”

An issue with calculating *R*_0_ is that it is not intrinsic to the biology of the infection (incubation period, infectiousness, recovery time, etc.), rather it is a phenomenological output of the biology of the infection and contact structure of the society [16]. When modeling *R*_0_, it is commonly obtained under the assumption of perfect mixing i.e., a given agent has equal likelihood of infecting each of the other agents in the model [39]. When aggregated over large communities on the scale of cities and states, this is widely held to be a reasonable assumption. However, our model of a small population—which has clustered, highly overlapping contact structure with sustained regular contact—is quite heterogeneous. These features allow for more infection spread than in a perfectly mixed network and consequently result in a larger *R*_0_. We note that Gressman and Peck use similar reasoning to justify their elevated choice of *R*_0_ = 3.8 [9]. The contact structure in their university COVID-19 model is also heterogeneous.

A natural way to estimate *R*_0_ is to seed the student population with *s* on-campus students in the exposed state. We then run the model and count the resulting number of direct infections *I*(*s*) that arise from these *s* agents. A sample of *R*_0_ from this seed is then computed via

$$\begin{array}{c} R_{0}\left(s\right)=\frac{I(s)-s}{s}. \end{array}$$ (4)

While *R*_0_(1) corresponds to the definition of the average reproduction number (minus the perfect mixing assumption), it is desirable to take *s* larger to smooth out the randomness arising during the agent’s progression through the infection and from their individual schedule.

**Fig 8:**
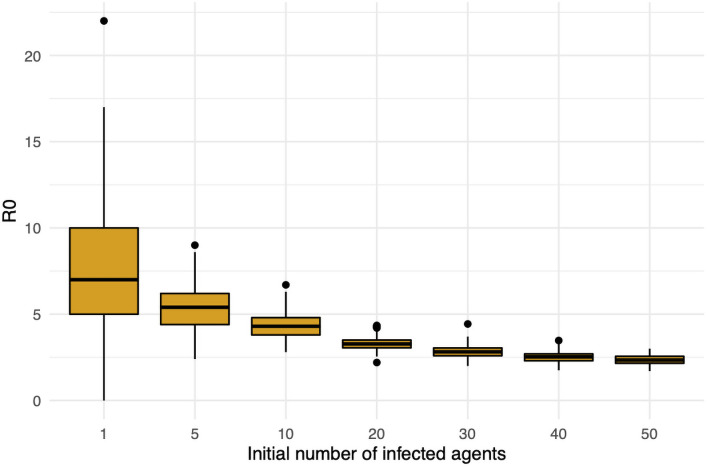
Empirical measurements of *R*_0_(*s*) computed as in (4) with different initial seed sizes *s* of the on-campus student population infected. The results from 100 runs are shown for each *R*_0_(*s*).

Such smoothing reveals a difficulty with measuring *R*_0_ in our model. Fig 8 shows significant variation in the *R*_0_ defined in (4). *R*_0_(1) ranges from 1 to 23 with mean 7.4. The value of *R*_0_(*s*) decreases quickly in *s*; it more than halves to have mean 3.33 at *s* = 20 and the mean drops below 2.35 for *s* ≥ 50. It is not obvious which value of *s*, if any, gives the “correct” *R*_0_. Note that this effect is a consequence of the contact structure in the model discussed in Section 2.4 and also the small total population of or model.

**Fig 9:**
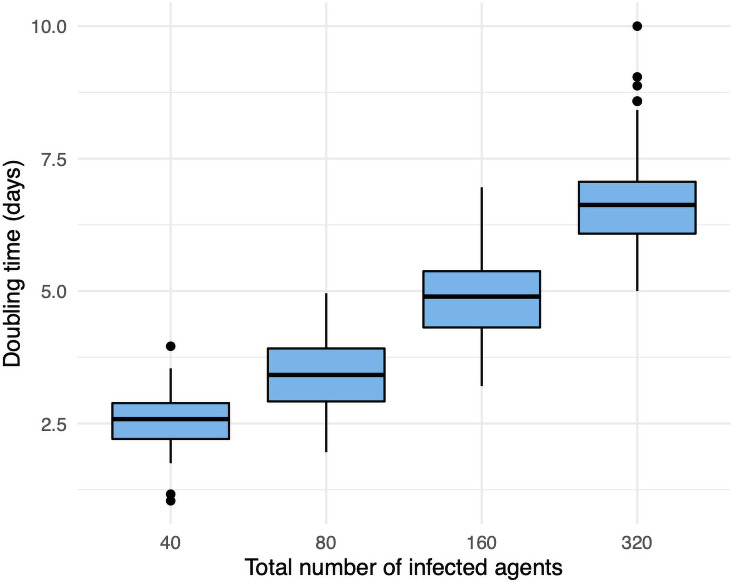
The average number of days (*y*-axis) to go from *x*/2 to at least *x* infections. We omit *x* = 20 since we initially seed 10 agents in the exposed state and there is latency for infections to begin. We omit *x* > 320 since for such large *x*-value the doubling time slows significantly from a herd-immunity effect.

The *doubling time* of the infection is another important statistic that is closely associated with *R*_0_ [39]. This is the average number of days for the number of total infections to double in an environment with no intervention. It is believed that the doubling time for COVID-19 lies in between 2 days and 4 days [40, 41]. In Fig 9 we display the average number of days for total infections to double in our base model. The average number of days to go from 20 infected agents to more than 40 is 2.5. The doubling time on the next interval [40, 80] is 3.43, [80, 160] is 4.9, and [160, 320] is 6.7. At this point 320/2380 ≈ 13% of the population is infected. Thus, the depleting population size is slowing infection spread. These doubling times are more compatible with an *R*_0_ in [2, 3], which is consistent with taking *s* ≥ 30 in (4).

In closing, the seed size and doubling time data suggests that measuring *R*_0_ in our model is subjective. Measuring intervention effectiveness through total infections against our Base Assumption is more transparent. Moreover, total infections are likely of greater help to policy makers since that data is directly available (via testing) rather than the inferred statistic *R*_0_.

### 4.2 Sensitivity to global parameters

**Fig 10:**
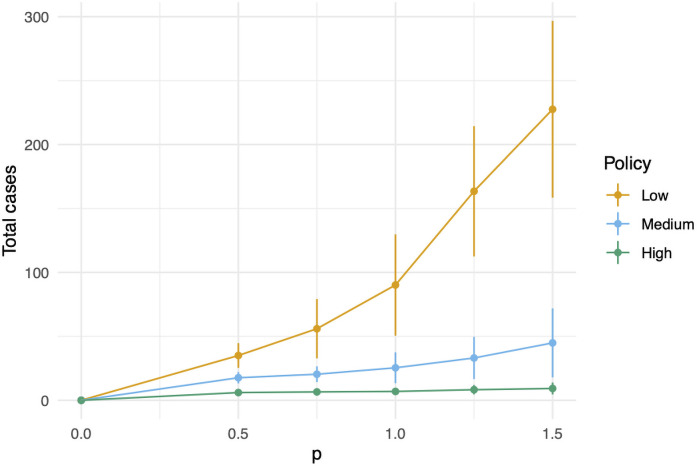
A sensitivity analysis of the tuning parameter, *p*. We fix the student adherence to be medium, and show the total number of cases for each of the three administrative policies.

In our model, there are two events in which susceptible agents may become infected: (i) interaction with an infected agent on campus and (ii) interaction with an infection arising off-campus. All infections from (i) occur from face-to-face interaction at a site of the network. Transmission is thus proportional to the risk of transmission at the vertex times the number of infected agents at vertex *v* at a particular day and time, scaled via a tuning parameter *p* (see (2)). In Fig 10 we vary *p* in {0.00, 0.25, …, 1.5}, given a fixed level of student compliance (medium) and varying policy intensity. Under these different scenarios, the relative effectiveness of the various policies remains roughly proportional.

**Fig 11:**
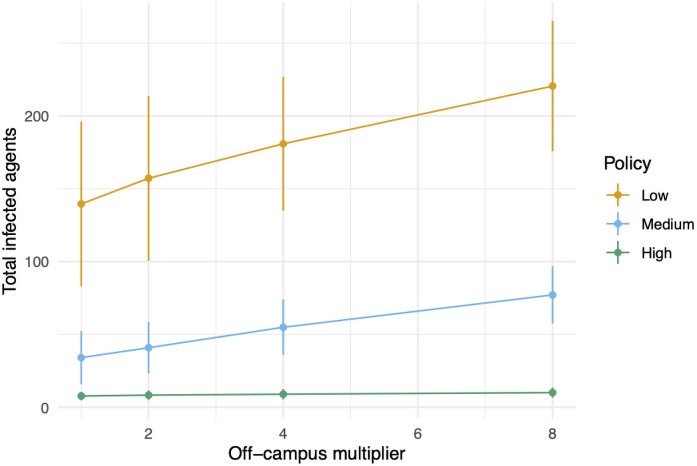
A sensitivity analysis of the off-campus multiplier. We fix the student adherence to be medium, and show the total number of cases for each of the three administrative policies.

The second pathway for infection is through exogenous infections arising off-campus. The base model has on average one new off-campus infection every two weeks. This comes from each of the *n*_0_ + *n*_*f*_ agents coming and going from campus probability

$$\begin{array}{c} o=\frac{0.125}{\left(n_{o}+n_{f}\right)} \end{array}$$

of becoming infected on a given instruction day. In Fig 11 we test the effect of multiplying *o* by a factor in {1, 2, 4, 8} on total infections with medium student adherence and varying policy intensity. We see that there is not much sensitivity to this choice. Increasing *o* by a factor of 8 (so there are on average 4 exogenous infections per week) does not significantly change the total number of new cases.

**Fig 12:**
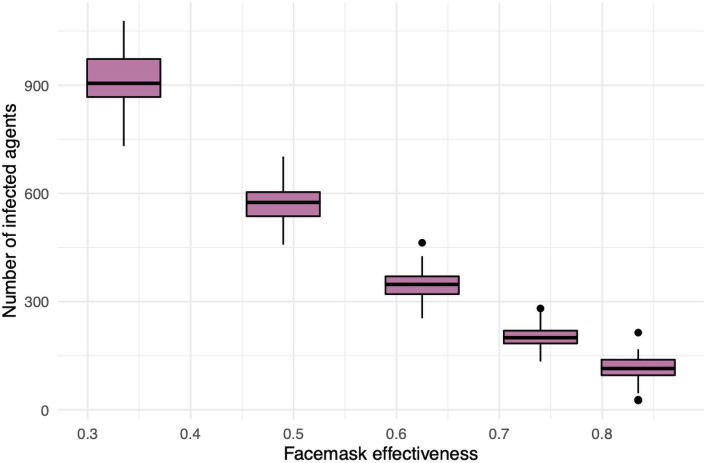
A sensitivity analysis of facemask effectiveness. Displayed are total number of infections after a semester with f=1 (perfect facemask compliance), but no other intervention.

Lastly, the single most effective intervention is facemask use (see Fig 1). Accordingly, we explore sensitivity to that feature. Recall that the parameters m and $m^{\prime }$ dictate the reductive factor for the probability of an infected facemask wearer infecting others (m) and a susceptible wearer becoming infected $\left(m^{\prime }\right)$. See (3). We call the quantity $M=1-m\cdot m^{\prime }$ the *facemask effectiveness* since it gives the reduction in transmission probability when both parties (infected and susceptible) are wearing facemasks. Our default choice is m=0.5 and *m*′ = 0.75 which gives *M* = 0.625. This is consistent with current estimates for facemask effectiveness [21–26]. Nonetheless, in Fig 12 we show the resulting number of total infections when f=1 and

$$\begin{array}{c} \left(m,m^{\prime }\right)\in \left\{\left(0.5,0.75\right)\pm n\left(0.1,0.1\right):n=-2,-1,0,1,2\right\}, \end{array}$$

so that *M* varies through the interval [0.335, 0.835]. What we observe is in line with the sensitivity analysis in Fig 10; facemask effectiveness has significant, yet predictable, impact on the total number of infections.

### 4.3 Future directions

A limitation of our model is that the way infections occur makes contact tracing impractical to implement. Unlike [9], in which contacts are known, we assume perfect mixing on the level of rooms, so it is not possible to infer who did the infecting. Staff and visitors to campus are another noteworthy feature that our model is missing. It would add more detail to include more variety in agent types and behavior and also consider other interventions as well as combined strategies. Introducing a vaccine to the infection dynamics could be useful.
